# Photographic evaluation of clinical activity score in thyroid eye disease

**DOI:** 10.1371/journal.pone.0337597

**Published:** 2025-12-12

**Authors:** Gyeong Min Lee, Min Joung Lee, Namju Kim, Hokyung Choung, Hwa Lee, Won-Kyung Cho, Jae Hoon Moon, Sang In Khwarg

**Affiliations:** 1 Department of Ophthalmology, Hallym University Kang Nam Sacred Heart Hospital, Seoul, Republic of Korea; 2 Department of Ophthalmology, Hallym University Sacred Heart Hospital, Anyang, Republic of Korea; 3 Department of Ophthalmology, Seoul National University Bundang Hospital, Seongnam, Republic of Korea; 4 Department of Ophthalmology, Seoul National University, College of Medicine, Seoul, Republic of Korea; 5 Department of Ophthalmology, SMG-SNU Boramae Medical Center, Seoul, Republic of Korea; 6 Department of Ophthalmology, Korea University Ansan Hospital, Ansan, Republic of Korea; 7 Department of Ophthalmology, Uijeongbu St. Mary’s Hospital, Uijeongbu, Republic of Korea; 8 Department of Internal Medicine, Seoul National University Bundang Hospital, Seongnam, Republic of Korea; 9 Department of Ophthalmology, Seoul National University Hospital, Seoul, Republic of Korea; Hangil Eye Hospital / Catholic Kwandong University College of Medicine, KOREA, REPUBLIC OF

## Abstract

**Purpose:**

Given the subjective nature of clinical activity score (CAS) and variability between observers, this study evaluated the inter-observer variability of soft tissue signs (STS) in the CAS using periocular photographs and compared the diagnostic performance of photographic CAS (P-CAS) with clinical assessment for detecting active thyroid eye disease (TED).

**Methods:**

This retrospective cohort study included 754 TED patients who underwent periocular photography at Seoul National University Bundang Hospital between 2006 and 2021. Five oculoplastic specialists independently evaluated five STS of CAS using periocular photographs. Inter-observer agreement and concordance between photographically (P-STS) and clinically (C-STS) assessed STS, assessment consistency, were analyzed using Kappa statistics. Diagnostic accuracy of P-CAS was evaluated via receiver operating characteristic (ROC) curves with additional analysis incorporating demographic variables such as sex, age, and smoking dose.

**Results:**

Assessment consistency was moderately good for redness of eyelid and conjunctiva (Kappa = 0.418, 0.499). The diagnostic performance of P-CAS (area under the curve, AUC = 0.774) was lower than that of clinical CAS (C-CAS) (AUC = 0.818, *p* = 0.007), However, it significantly improved when demographic factors were added (AUC = 0.851).

**Conclusion:**

Periocular photography provides a reproducible and clinically valuable adjunct for assessing TED activity. The redness-related items showed higher agreement than swelling-related ones, and integrating demographic variables enhances diagnostic performance. This approach may contribute to standardizing TED assessment and serve as a foundation for future automated or image-based evaluation tools.

## 1. Introduction

Thyroid eye disease (TED), also called Graves’ ophthalmopathy, is an autoimmune disease of the orbit characterized by signs of periocular inflammation and soft tissue congestion [1]. The natural course of TED presents with a progressive, active phase, followed by a stable, inactive phase. It is necessary to determine the disease phase, since the response to immunosuppressive treatment is better in the active phase of TED than that in the chronic inactive phase [2–4].

The clinical activity score (CAS), devised by Mourits et al. [4] is an assessment method that identifies the activity of TED by summing the scores of the periocular inflammatory features. At the initial visit, the CAS is determined using 7 symptoms and signs of periocular soft tissue inflammation scored on a binary scale, with each parameter being weighted equally. The scores are rated on a 7-point scale: 5 points are assigned to objective periocular signs of inflammation and 2 points to subjective pain symptoms. A score of 3 or higher is considered to be indicative of active TED [5].

The CAS is easy to use and is one of the most commonly used scoring system for assessing TED activity. Despite its popularity, it has a few limitations. CAS uses a binary scale where each item is given equal weight, and it includes subjective symptom items. Even when assessing periocular soft tissue signs, the clinician’s subjective judgment influences the scoring. However, variability among clinicians has rarely been evaluated [6].

Periocular photographs are easy to obtain and objectify and can also serve as a record of the patient’s progress and therapeutic effect. They can be also used to analyze the difference in the CAS according to the measurer. Moreover, photographic assessment of the CAS has garnered importance with the advancement of telemedicine, contactless clinics, and the use of artificial intelligence in the medical field. Thus, we conducted a large-scale analysis of assessing the inter-observer agreement of the photographic CAS (P-CAS) and its agreement with the clinical CAS (C-CAS). The diagnostic ability of P-CAS for differentiating between active and inactive TED was also analyzed.

## 2. Materials and methods

### Study population

This study enrolled patients aged 18 years or older with TED who visited Seoul National University Bundang Hospital from January 2006 to March 2021. The medical records and digital photographic images (DPIs) of the eyes at the initial visit were analyzed retrospectively. Patients without DPIs, CAS evaluation, or those diagnosed with other concomitant ophthalmic or periocular diseases were excluded. The Institutional Review Board of Seoul National University Bundang Hospital (IRB# B-2208-775-106) approved this study, which was conducted in accordance with the tenets of the Declaration of Helsinki. The institutional review board waived the requirement for informed consent since the study design was based on a retrospective review of medical records and pre-obtained facial images. The data were accessed for research purposes on September 06, 2022. All data used in this study were de-identified prior to analysis, and the authors did not have access to any information that could identify individual participants.

### Collection of clinical data

We collected patients’ data including age, sex, and smoking history, and the CAS. The modified CAS, which was used to assess TED activity, consists of 10 items representing 2 classic inflammation symptoms, 5 soft tissue signs, and 3 measurements suggesting progression: 1) spontaneous pain behind the globe, 2) pain on attempted gaze, 3) redness of eyelid, 4) conjunctival injection, 5) swelling of caruncle, 6) swelling of eyelid, 7) chemosis, 8) increase in proptosis, 9) decrease in ocular excursion, and 10) decrease in visual acuity. CAS scoring is binary for each item. A CAS ≥ 3 at the initial visit or CAS ≥ 4 at the follow-up visit within 3 months was defined as active TED [7].

### Acquisition of DPIs

DPIs were acquired in a studio environment using identical camera settings under standardized lighting, using standardized and reproducible techniques. The patients’ digital eyelid images were obtained from a distance of 1 meter using a digital single-lens reflex camera (Canon EOS 40D, 2976 × 1984 resolution, Tokyo, Japan). The patient was instructed to look directly at the camera with the eyelids open, eyebrows relaxed, and a level head. The DPIs were processed such that the eye periphery was visible with the eyebrow as the upper boundary and the pupillary reflex point as the center.

### Photographic soft tissue signs

Five oculoplastic specialists, each with > 15 years of clinical experience, investigated the same DPIs and rated five CAS parameters to facilitate analysis of the inter-observer agreement of each parameter. Thereafter, in order to achieve a consensual photographic evaluation result in the event of any discrepancies, an agreement was reached through discussion, and the agreed score for each soft tissue item was defined as the photographic soft tissue sign (P-STS). The correspondence between the P-STS and the clinical soft tissue signs (C-STS) in the medical records was analyzed.

### Photographic CAS

The scores of 5 P-STS items and the 2 subjective items of the CAS were added to calculate the P-CAS. We assessed the sensitivity and specificity of the P-CAS for discriminating active and inactive TED patients. The diagnostic performance of the P-CAS was also compared to that of the C-CAS.

### Statistical analysis

Statistical analysis was performed using SPSS for Windows version 27 (SPSS Inc., Chicago, IL, USA) or R version 4.1.3. Statistical significance was set as p < 0.05. Fleiss’ kappa was calculated to evaluate inter-observer agreement. The agreement between P-STS and C-STS was calculated with Cohen’s kappa coefficient. The threshold for kappa was set at 0.4, corresponding to a moderate level of reliability, according to the Landis & Koch scale (kappa: < 0.20 slight, 0.21–0.40 fair, 0.41–0.60 moderate, 0.61–0.80 substantial, 0.81–1.00 almost perfect) [8]. Logistic regression analysis was used to model the predictive probability of diagnostic performance by adding clinical factors to the P-CAS. Diagnostic performance was evaluated using receiver operating characteristic (ROC) curve analysis, and the significance of the area under the curve (AUC) was determined using the paired ROC comparison test (Delong’s test) [9]. Descriptive statistics were calculated for all demographic and clinical variables. Chi-squared tests were used for categorical variables, and continuous variables were analyzed using the t-test.

## 3. Results

### Characteristics of the study population

We reviewed medical records of a total of 921 patients with TED and 754 patients fulfilled the inclusion criteria. We excluded 167 patients from the study (incomplete clinical records, n = 116; missing photographic data, n = 44; other concomitant ophthalmic diseases, n = 7).

### Inter-observer agreement of the P-STS

**Fig 1:**
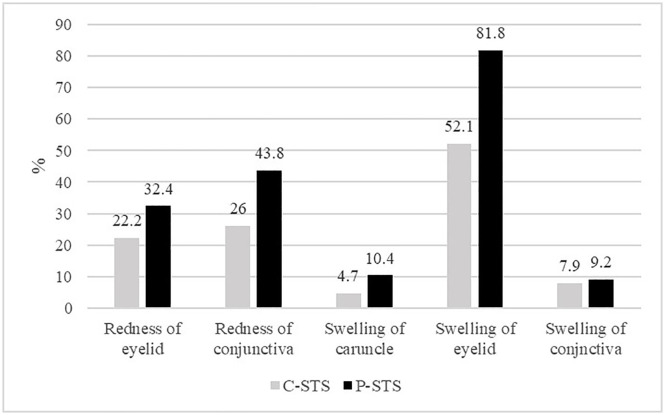
Positivity rates of the photographic and clinical soft tissue parameters P-STS: photographic soft tissue signs, C-STS: clinical soft tissue signs.

The inter-observer agreement for 5 P-STS parameters was at least fair (kappa>0.2), and moderately agreement (kappa>0.4) for redness of the eyelid and conjunctival injection. The agreement between the P-STS and C-STS was also at least fair, and moderately good for redness of the eyelid, conjunctival injection, and swelling of the conjunctiva (Table 1). The positivity rate of the P-STS was higher for all parameters compared to C-STS, especially for eyelid swelling, where the positivity rate of the P-STS was the highest (Fig 1).

**Table 1:** Inter-observer agreement for the P-STS and agreement between the P-STS and C-STS.

	Inter-observer agreement of P-STS		Agreement between P-STS and C-STS	
	Kappa	Interpretation	Kappa	Interpretation
Redness of eyelid	0.418	Moderate	0.431	Moderate
Redness of conjunctiva	0.499	Moderate	0.444	Moderate
Swelling of caruncle	0.230	Fair	0.355	Fair
Swelling of eyelid	0.366	Fair	0.230	Fair
Swelling of conjunctiva	0.286	Fair	0.404	Moderate

P-STS: photographic soft tissue signs, C-STS: clinical soft tissue signs

### Clinical risk factors associated with TED activity

We divided the study population (n = 754) into 2 groups according to disease activity to investigate the factors associated with active TED (Table 2). The inactive TED group consisted of 527(69.9%) patients and the active TED group consisted of 227(30.1%) patients. Although the proportion of women was predominant in both groups, the proportion of men in the active TED group was significantly higher than that in the inactive TED group (*p* < 0.001). The mean age of the active TED group was significantly higher than that of the inactive TED group by 11.39 years (*p* < 0.001). Smoking history was available for 718 patients; the proportion of patients with smoking history was significantly higher in the active TED group than that in the inactive TED group (p < 0.001). The average number of pack-years of smoking was higher by 6.64 in the active TED group than that in the inactive TED group (*p* < 0.001). The P-CAS and C-CAS were also higher in the active TED group than those in the inactive TED group. The sensitivity and specificity of the P-CAS was 61% and 82.5%, respectively, with a cut-off value of 3. The sensitivity of C-CAS was 52.9% and the specificity was 94.9%.

**Table 2:** Comparison of the clinical characteristics between the groups with active and inactive TED.

Characteristics	Inactive TED (n= 527)	Active TED (n=227)	*P*
Sex			
Male, n (%)	132 (25.0)	131 (42.3)	<0.001
Mean age (years) ± SD	41.16 ± 14.76	52.55 ± 12.89	<0.001
Smoking status, n (%)			<0.001
Never smoker	315 (41.8)	109 (14.5)	
Ever smoker	180 (23.9)	114 (15.1)	
NA (%)	32 (4.2)	4 (0.5)	
Smoking Intensity			
PY, mean ± SD	4.03 ± 8.95	10.67 ± 18.94	<0.001
PY range, n (%)			<0.001
0 ≤ PY < 1	347 (70.1)	115 (51.6)	
1 ≤ PY < 10	66 (13.3)	34 (15.2)	
10 ≤ PY <20	38 (7.7)	21 (9.4)	
PY ≥ 20	44 (8.9)	53 (23.8)	
P-CAS	1.61 ± 1.03	2.94 ± 1.33	<0.001
C-CAS	0.87 ± 0.95	2.56 ± 1.51	<0.001

CAS: clinical activity score, P-CAS: photographic CAS, C-CAS: clinical CAS, PY: pack-years, TED: thyroid eye disease

### Diagnostic performance of P-CAS and C-CAS for assessing active TED

**Fig 2:**
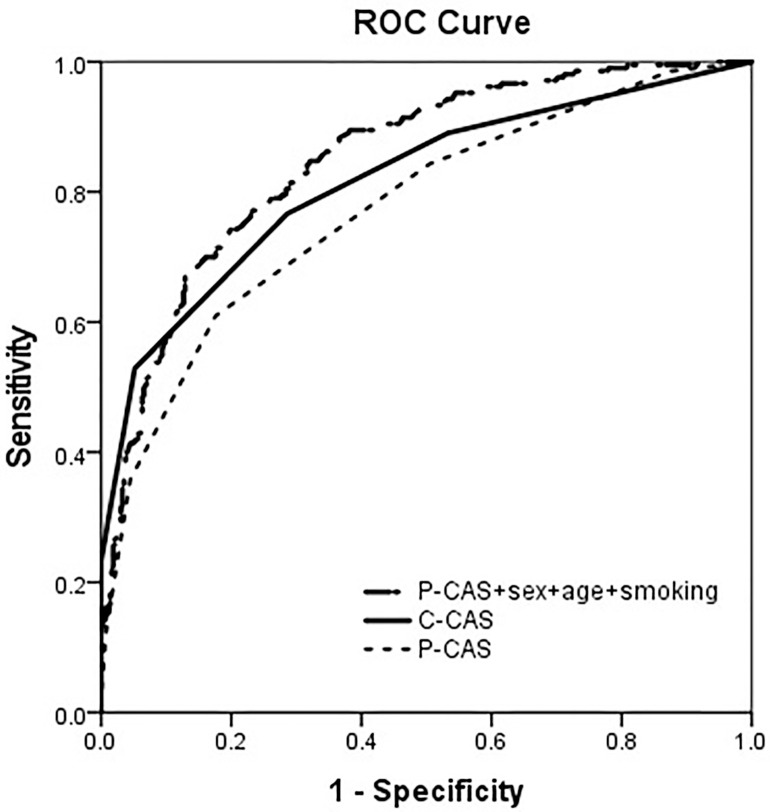
Receiver operating characteristic curves of P-CAS, C-CAS, and P-CAS combined with sex, age, and smoking for differentiating between active TED and inactive TED patients. CAS: clinical activity score, P-CAS: photographic CAS, C-CAS: clinical CAS, TED: thyroid eye disease.

The AUC of P-CAS was significantly lower than that of C-CAS (0.774 vs 0.818, *p* = 0.007, Delong’s test). However, the addition of demographic factors to the P-CAS enhanced the diagnostic performance compared to that of P-CAS alone. The addition of sex, age, and smoking to P-CAS resulted in a higher AUC than that of the C-CAS and attained borderline significance (Fig 2 (AUC = 0.851, *p* = 0.064) and Table 3).

**Table 3:** Area under the ROC curve of P-CAS with other clinical factors for predicting thyroid eye disease activity.

Prognostic factor	AUC	95% CI	p-value compared to P-CAS	p-value compared to C-CAS
P-CAS	0.774	0.735-0.814		0.007
C-CAS	0.818	0.781-0.855	0.007	
P-CAS with clinical factors				
P-CAS with sex	0.792	0.754-0.830	<0.001	0.116
P-CAS with age	0.835	0.802-0.868	<0.001	0.337
P-CAS with smoking	0.804	0.767-0.841	0.001	0.438
P-CAS with sex, age	0.848	0.816-0.879	<0.001	0.096
P-CAS with sex, smoking	0.808	0.772-0.845	<0.001	0.595
P-CAS with age, smoking	0.846	0.815-0.878	<0.001	0.105
P-CAS with sex, age, smoking	0.851	0.820-0.881	<0.001	0.064

ROC curve: receiver operating characteristic curve, CAS: clinical activity score, P-CAS: photographic CAS, C-CAS: clinical CAS

## 4. Discussion

We demonstrated that periocular photography can be an adjuvant method for evaluating the CAS. P-CAS, which was derived from consensus-based soft tissue sign score, was comparable with C-CAS when combined with basic demographic features.

The CAS is the primary method employed to evaluate TED activity in clinical practice. However, the need for objective methods to assess the CAS or alternative methods to assess TED activity has arisen, since the CAS can be affected by the subjective judgment of the observer, leading to inter-observer variability, as demonstrated by a recent British study [10]. Thus, we sought to compare the degree of concordance between observers using periocular photographs. We investigated the inter-observer agreement of the P-STS using DPIs, along with the agreement between the C-STS and P-STS. All P-STS parameters showed inadequate inter-observer agreement. The eyelid and conjunctiva redness showed moderate inter-observer agreement, whereas the eyelid swelling, conjunctival swelling, and swelling of caruncle showed fair inter-observer agreement. The redness-related parameters (redness of the eyelid and conjunctiva) showed higher agreement than swelling-related parameters (swelling of the eyelid, conjunctiva, and caruncle), probably because color perception is more intuitive than shape perception [11,12]. Although all photographs were obtained under a standardized protocol, redness still appeared more pronounced in photographs than in clinical examination, likely due to red-channel overemphasis by digital sensors. The use of color calibration tools might help minimize systematic overestimation of redness and improve the accuracy, although further studies are needed to confirm its effect. Perros et al. [10] recently demonstrated that the eyelid parameters (swelling and redness of the eyelid) showed lower inter-observer agreement than the conjunctival parameters (swelling and redness of the conjunctiva). However, it is difficult to compare the results of that study with ours, as the former examined patients face-to-face, included a small patient population, and used different methods for statistical analysis. Another multicenter study [6] analyzed the intra-rater and inter-rater reliability of C-STS assessment by two observers. Although that study did not perform photographic analysis, conjunctival redness showed higher intra- and inter-observer accuracy than conjunctival swelling, similar to our results.

Furthermore, all P-STS parameters showed a higher positivity rate compared to the C-STS parameters, so the P-STS score tends to be higher than the C-STS score. It is probably because the characteristics of the P-STS parameters on the DPIs are enhanced to a greater degree than reality. Eyelid edema showed the highest positivity rate among the P-STS parameters, probably because it was difficult to discriminate between the pre-existing natural puffiness and inflammatory swelling using DPIs in an Asian population [13]. In addition, the two-dimensional nature of photographs results in loss of depth perception, which may accentuate periocular fullness and lead to overestimation of swelling compared with clinical examination [14].

In this study, P-CAS showed lower diagnostic ability than C-CAS. Then, we added demographic variables including age, sex, and smoking history which are known to be associated with TED activity. We found that the combination of P-CAS with these demographic factors substantially enhanced the diagnostic performance over that of P-CAS alone. P-CAS combined with age, sex, and smoking demonstrated the highest AUC (0.851), which was higher than that of the C-CAS (0.818), and attained borderline significance. These results suggest that P-CAS is less accurate than C-CAS, but the gap in diagnostic performance can be reduced by incorporating basic demographic factors. In practice, such a combined model may be used as a screening tool, especially in telemedicine, to help identify higher-risk patients for timely referral. It could also serve as a basis for AI tools that combine photographic and demographic data to improve TED activity assessment.

In the current study, the risk factors for active TED were male sex, old age and smoking dose, consistent with previous studies [15]. However, since sex, age, and smoking dose were not independent variables, we performed subgroup analysis according to age. In the younger group, sex, age, and smoking dose were associated with active TED, whereas in the older group, only age and smoking dose were significantly associated with active TED (S1 Table). The effect of sex appeared to decrease with increasing age and accumulation of smokers in the older group. Conversely, in the younger group, the effect of sex was the greatest, and the effect of age and smoking was small. Thus, younger men and older adults with a long history of smoking are more likely to have active TED.

This study has a limitation. Since this study was conducted at a single center and in a single ethnic group (Koreans), which may limit generalizability. Ethnic differences in eyelid anatomy, skin pigmentation, and baseline ocular redness could also affect P-CAS interpretation, underscoring the need for validation in multi-center, multi-ethnic cohorts. The P-CAS was evaluated solely based on the frontal photograph. Multidimensional photographs and slit-lamp images would facilitate a more accurate assessment of the chemosis and caruncle swelling. Because two-dimensional photographs lack depth information, swelling may be overestimated. Future use of three-dimensional imaging could provide a more accurate evaluation of these parameters [14].

In conclusion, inter-observer agreement of P-STS was limited, particularly for swelling-related items which showed only fair agreement. These results suggest the need for careful evaluation of soft tissue signs in photographs and underscore the importance of reaching a consensus among multiple clinicians. In this study, P-CAS achieved diagnostic accuracy comparable with C-CAS when combined with sex, age, and the smoking dose. This suggests that P-CAS (CAS assessment using photographs), when integrated with basic demographic data, can serve as a clinical tool, and may also provide a foundation for future AI-based automated evaluation. To the best of our knowledge, this is the largest case series to date evaluating the periocular photographs of patients with TED. Future research should focus on developing multimodal technologies capable of quantitatively measuring CAS to enhance diagnostic and treatment precision.

## Supporting information

S1 Table Risk factor analyses for active TED stratified by age group. (DOCX)

## Abbreviations

AUC: area under the curve
TED: thyroid eye disease
STS: soft tissue signs
CAS: clinical activity score
DPI: digital photographic image
P-STS: photographic soft tissue sign
C-STS: clinical soft tissue sign
P-CAS: photographic CAS
C-CAS: clinical CAS
ROC: receiver operating characteristic
