# Supplementary material for: Photographic evaluation of clinical activity score in thyroid eye disease
Source: PLoS One. 2025 Dec 12;20(12):e0337597. doi: 10.1371/journal.pone.0337597 (PMC12700362; doi:10.1371/journal.pone.0337597)
Supplement: S1 Table — (DOCX) [file pone.0337597.s001.docx]

**Supplementary Table 1. Risk factor analyses for active TED stratified by age group**

|  | Group 1  Age<50 years (n=430) | | | | | |  | Group 2  Age ≥50 years (n=288) | | | | | | |
| --- | --- | --- | --- | --- | --- | --- | --- | --- | --- | --- | --- | --- | --- | --- |
|  |  | | Male  (n = 134) | | Female  (n = 296) | |  |  | | Male  (n = 86) | | Female  (n = 202) | | |
| Variables | OR  (95% CI) | P | OR  (95% CI) | P | OR  (95% CI) | P |  | OR  (95% CI) | P | OR  (95% CI) | P | OR  (95% CI) | P |  |
| Sex (Male:Female) | 1.84  (1.02-3.30) | **0.042**^*^ |  |  |  |  |  | 1.08  (0.56-2.08) | 0.816 |  |  |  |  |  |
| Age | 1.05  (1.02-1.08) | **0.002**^*^ | 1.05  (0.99-1.11) | 0.086 | 1.05  (1.01-1.09) | **0.011**^*^ |  | 1.05  (1.02-1.09) | **0.003**^*^ | 1.05  (0.99-1.11) | 0.111 | 1.05  (1.01-1.09) | **0.016*** |  |
| Smoking (pack-years) | 1.03  (1.00-1.06) | **0.033**^*^ | 1.03  (0.99-1.06) | 0.129 | 1.06  (0.99-1.14) | 0.084 |  | 1.02  (1.00-1.04) | **0.045**^*^ | 1.04  (1.01-1.07) | **0.015**^*^ | 0.99  (0.95-1.03) | 0.580 |  |
|  |  |  |  |  |  |  |  |  |  |  |  |  |  |  |

TED: thyroid eye disease
